# Comprehensive psychometric examination of the attitudes towards suicide (ATTS) in South Korea

**DOI:** 10.1186/s13033-016-0035-0

**Published:** 2016-01-16

**Authors:** Nam-Ju Ji, Yeon-Pyo Hong, Weon-Young Lee

**Affiliations:** Department of Preventive Medicine, Chung-Ang University College of Medicine, 84 Heukseok-Ro, Dongjak-Gu, Seoul, 156-756 Republic of Korea

**Keywords:** Attitude to death, Suicide, Reliability and validity, Prevention, Instruments

## Abstract

**Background:**

The aim of this study is to examine the validity and reliability of the Korean version of attitudes towards suicide (ATTS) on a group of university students, which would contribute to the evaluation of the ATTS as a useful tool of measuring attitudes toward suicide in South Korea with very high suicide rates.

**Methods:**

The subjects of the study were 195 undergraduates at Chung-Ang University, South Korea in 2013. The measure for assessing public attitudes towards suicide was ATTS made up of 34 items in English and the Korean version of it was produced by forward and backward translation procedure. To identify any factors unique to South Koreans’ attitudes towards suicide, we applied exploratory factor analysis (EFA) to the data from 195 university students and was followed by confirmatory factor analysis (CFA) to assess construct validity of the Korean version. The internal consistency of the scale was assessed using Cronbach’s α and the assessment of the test–retest reliability was performed by intraclass correlation coefficients.

**Results:**

On the EFA analysis, were excluded, the tool had 11 factors (32 items), accounting for 62.99 % of the total variance in participants’ responses. CFA failed to support 11-factor model of the scale. Six out of 11 factors were acceptable in terms of both internal consistency and test–retest reliability.

**Conclusions:**

Six factors of the Korean version of the ATTS had acceptable content validity and reliability. However, on the whole, it did not have good construct validity and thus further investigations are needed to develop a scale measuring true public attitudes toward suicide in South Korea.

## Background

Measuring the attitudes toward suicide has been a long term interest for those who have been specializing in suicide [1–5]. ‘Attitudes toward suicide (ATTS)’ is influenced not only by an individual factor such as the character, sex, age, residential types, experience related to suicide, contact with a suicidal person, personal beliefs and so on but also by culture, social role and even by the instrument used to measure it [6–14]. As the attitude is influenced by diverse factors, it is extremely difficult to measure attitude for suicide accurately.

Nevertheless, this is a very important matter because by being able to measure the attitude, we can find vulnerable people to suicide and a group or community requiring suicide prevention. And also, measuring of attitude toward suicide could be an evaluation tool for the effectiveness of an suicide prevention education program and helps to develop an community suicide prevention program through improved awareness of suicide [1, 2].

Among them, the most representative and widely used tool is the following three: suicide opinion questionnaire (SOQ), the suicide attitude questionnaire (SUIATT) and ATTS [2]. Of them, we chose ATTS to look for an appropriate scale measuring South Koreans’ attitudes towards suicide. A psychometric test of the Korean ATTS would be added to the evidence from a few studies regarding that of ATTS [10, 15, 16].

### Aims

The aim of this study is to examine the validity and reliability of the ATTS on a group of university students, which would contribute to the evaluation of ATTS as a useful tool of measuring attitudes toward suicide in South Korea with very high suicide rates.

## Methods

### Subjects

The target subjects of the study were 241 undergraduates, including 105 pre-medicine undergraduates who took the cultural subject course “Modern Society and Health,” at Chung-Ang University, Seoul, Republic of Korea in 2013. 195 students (80.9 %) of the total subject population agreed to fill out a self-reported questionnaire, whereas 46 undergraduate students were unable to participate as they were in absence from class. The questionnaire included 34 items from the ATTS and were added 4 demographic items such as sex, age, major and the university year. The Institutional Review Board of Chung-Ang University approved the protocol, survey instruments, and consent document for this study (IRB no. 1041078-201311-HR-0093-02).

### Instrument and procedure

The ATTS was developed through two survey studies conducted in 1986 and 1996 [16]. The first version of ATTS was developed in 1986 based on the SOQ [5] and was expanded in the second 1996 study to reflect related atti-tudinal studies. ATTS was predominantly used in Europe to measure the attitudes toward suicide [16, 17], but in recent years has been used globally including within Asian countries such as South Korea and Japan [8, 10, 11, 13, 18], as well as Africa [9, 19] and others. In this study, we adopted the second version of ATTS which has better validity and reliability than its first version [16].

Exploratory factor analysis (EFA) of ATTS version 2 yielded 10 interpret-able factors, which accounts for 60 % of the total variance and Cronbach’s *α* coefficients for those factors ranged from 0.38 to 0.86 [2, 8, 10, 11, 14–16].

The ver-sion 2 in 1996 contained a total of 34 items [16]. These were scored on a five-point Likert scale (agree completely, agree to a large extent, doubtful, do not agree and agree not at all). For easier comprehension, this study applied a reversed scoring system. For example, the participant’s answer of “agree completely” to the item “Suicide understandable if severe, incurable disease-people” would correspond to the minimum score of one according to the original scoring system, but is scored the maximum of five in this study. Therefore the stronger the participant felt about a particular subject, the higher their score would become.

For this study, the original second version of ATTS with 34 items was translated into Korean using a forward and backward translation. The process for a forward–backward translation of ATTS and its psychometric testing, which occurred between November 16 and December 2, 2013.

Firstly, the forward translation of the English version of the ATTS into Korean was undertaken by a psychologist and a psychiatrist. Then, the researchers reviewed these two versions and reached a consensus on the Korean draft version. Secondly, a Korean–Australian bilingual expert, translated the draft back into English. The translator and the researchers compared the backward-translated English version with the original one in terms of conceptual equivalence, and then the Korean version was revised slightly. Through this process, the Korean version was completed and made available for the validity and reliability assessment.

### Statistical analyses

To identify any factors unique to Koreans’ attitudes, EFA was performed with the Korean version of ATTS and then, confirmatory factor analysis (CFA) applied with the Korean version using the number of factors resulted from the EFA earlier, to examine its structural validity. We performed EFA to extract factors using the principal component analysis (PCA) with promax rotation and factor loading >0.4 on each item was considered to belong to the corresponding factors [20]. The internal consistency of the preliminary version of ATTS was assessed using Cronbach’s α, and intraclass correlation (ICC) coefficients was used to assess test–retest reliability. ICC coefficients were interpreted with the following criteria: ICC coefficients <0.4, poor; 0.4< ICC coefficients <0.75, fair or good, ICC coefficients >0.75, excellent [21]. The assessment of the test–retest reliability was performed by administrating the questionnaire after a month to 71 consenting pre-medical undergraduate students. A 1 month interval was deemed sufficient since it was greater than the minimum of 2 weeks required for a wash-out effect of the previous questionnaire [22].

For CFA analysis, indices that were used to assess the fit of the model included: (1) X^2^-value/degree of freedom (df); (2) normed fit index (NFI); (3) Tucker-Lewis index (TLI); (4) Parsimony Normed Fit index (PNFI); (5) Root mean square error of approximation (RMSEA). With large samples, X^2^/df ratio of two or less is recommended for interpreting good fit. Cut-off value for interpreting good fit for the NFI and the TLI are 0.90. The recommended cut-off value for the PNFI is 0.80. Recommendations for interpreting fit for the RMSEA are <0.05 for good fit and between 0.05 and 0.08 as adequate fit [23, 24].

All analyses were performed using IBM’s SPSS Statistics 20.0 and AMOS 21.0 for Windows. A *p* value of under 0.05 was considered statistically significant.

## Results

### Participant demographics

The general demographical characteristics of the study subjects are shown in (Table 1). In a total of 195 participants, approximately 17 % more men (58.5 %) participated than women (41.5 %). Most participants (58.5 %) were between 20 and 24 years of age, and the proportions of first, second, third, and fourth-year students were 45.1, 35.9, 5.6, and 13.3 %, respectively. Pre-medical students accounted for 46.2 %, social science for 20.0 %, natural science for 20.5 %, and art and human science for 13.3 % (Table 1).

**Table 1:** General characteristics of study subject

Characteristics	N (%)
Gender	
Male	114 (58.5)
Female	81 (41.5)
Age (year)	
≤19	64 (32.8)
20–24	114 (58.5)
≥25	17 (8.7)
School year	
First	88 (45.1)
Second	70 (35.9)
Third	11 (5.6)
Fourth	26 (13.3)
Major	
Medical science	90 (46.2)
Social science	39 (20.0)
Natural science	40 (20.5)
Art and human science	26 (13.3)

Unit: person (%)

### Construct validity

As shown in Table 2, after the exclusion of two items with factor loadings <0.4, EFA revealed the following 11 interpretable factors: (1) Acceptability (2) Acceptability related incurable disease (3) Preventability (4) Tabooing (5) Unpredictability (6) Normal–common (7) Aging (8) Incomprehensibility (9) Suicidal process (10) Non-communication (11) Relation-caused (Table 2).

**Table 2:** Factors obtained from exploratory factor analysis and internal consistency for the ATTS

	E.V.	F.L.	Cronbach’s α	ICC coefficients
Total	62.99		0.657	0.782
Factor 1. Acceptability	15.709		0.762	0.832
Right to commit suicide—people		0.785		
Suicide should not always be prevented		0.627		
Suicide a relief		0.622		
Suicide can never be justified		−0.607		
Not understandable that people can take their lives		−0.606		
Most people avoid talking about suicide		0.414		
Factor 2. Acceptability related incurable disease	8.232		0.781	0.750
Get help to commit suicide if severe, incurable disease—myself		0.902		
Consider suicide if severe, incurable disease—myself		0.828		
Get help to commit suicide if severe, incurable disease—people		0.598		
Suicide acceptable means to end incurable disease—people		0.521		
Suicide understandable if severe, incurable disease—people		0.514		
Factor 3. Preventability	6.253		0.530	0.771
Can always help		0.882		
Suicide can be prevented		0.659		
Duty to restrain a suicidal act		0.506		
Factor 4. Tabooing	5.486		0.560	0.570
Risk to evoke suicidal thoughts if asked about		0.875		
Should or would rather not talk about suicide		0.640		
Suicide decision can’t reversed		0.470		
Factor 5. Unpredictability	4.8854		0.497	0.671
Relatives have no idea about what is going on		0.764		
Suicide happens without warning		0.694		
People who make threats seldom complete suicide		0.436		
Factor 6. Normal–common	4.708		0.516	0.758
Everyone has considered suicide		0.810		
Anybody can commit suicide		0.727		
Could express suicide wish without meaning it- myself		0.520		
Factor 7. Aging	4.109		1.0	0.576
Suicides among younger people particularly puzzling		0.926		
Factor 8. Incomprehensibility	3.620		0.339	0.387
Communication not serious		0.799		
Suicide among the worst thing to do to relatives		0.497		
Factor 9. Suicidal process	3.499		0.380	0.748
Suicides considered for a long time		0.971		
Attempts are impulsive actions		−0.644		
Factor 10. Non-communication	3.434		0.309	0.533
Low estimated own suicide probability		0.795		
Suicide ones own business		0.422		
Factor 11. Relation-caused	3.056		0.364	0.354
Attempts due to revenge and punishment		0.810		
Attempts due to interpersonal conflicts		0.703		

The 11 factors explained 62.9 % of the total variance and contained 32 of 34 items. Factor 1 and Factor 2 consist of items on the acceptability of suicide. Factor 2 focuses on the questions of the acceptability of suicide in those with incurable disease on two separate levels, one as the subject, that is myself or as the third person who is assumed to suffer from an incurable disease with the question raised being whether suicide is acceptable. Factor 3 is a section on the preventability of suicide and Factor 4 is composed of items related to the taboo of suicide: ‘Should or would rather not talk about suicide’, ‘Risk to evoke suicidal thoughts if asked about’. Factor 5 describes the unpredictability of suicide and Factor 6 consist of questions about whether suicide is Normal–common. The results of EFA analysis with Factor and their respective items are detailed in (Table 2).

#### Confirmatory factor analysis

After EFA, we performed CFA again with 11-factor model from the results of the EFA in our sample (Table 3). The CFA result value calculated by each of the indicators is as follows: X^2^/df = 1.93, NFI = 0.57, TLI = 0.63, PNFI = 0.44 and RMSEA = 0.07. With the exception of RMSEA, fit statistics were still below recommended cutoff values indicating the degree of fit was poor despite the slightly improved TLI comparing with the results of CFA using 10 factors shown in original version. Therefore, structural validity of the Korean version of ATTS is not good for the given data.

**Table 3:** CFA Results for Korean version of ATTS

Model	X^2^	df	X^2^/df	NFI	TLI	PNFI	RMSEA	90 % CI
Korean version of ATTS—11 factor model	790.35^a^	410	1.93	0.57	0.63	0.44	0.07	(0.06–0.08)

^a^Indicates statistically significant values at p < 0.001

### Reliability

The Cronbach’s α for the Korean version of the ATTS is 0.657 which being greater than 0.6, can be interpreted as having acceptable internal consistency as according to the general consensus on the cutoff value. Cronbach’s α values for the total 32 items ranged between 0.309 and 0.781, with all factors’ Cronbach’s α value greater than 0.3. (Table 2).

Factor 2 which comprises of items on acceptability related to incurable diseases had the highest value of 0.781, and Factor 1 which comprises of items on whether suicide is acceptable followed with 0.762. Factor 4 is composed of items related to the taboo of suicide followed with 0.560, then Factor 3 is a section on the preventability of suicide followed with 0.530. Factor 6 had Cronbach’s α of 0.516 and Factor 5 had Cronbach’s α of 0.497. Factor 7 contained 1 item that related to Aging followed with 1.0, Factor 9 with Cronbach’s α of 0.380, Factor 11 with Cronbach’s α of 0.364 and Factor 8 with Cronbach’s α of 0.339. Factor 10 had the lowest Cronbach’s α of 0.309.

The Korean version of the ATTS showed an excellent overall test–retest reliability, with an ICC coefficients of 0.782 (*p* < 0.001) and ICC coefficients values greater than 0.7 for 5 out of 11 factors (Factor 1–3, 6 and 9). The highest test–retest reliability was shown in Factor 1 with ICC coefficients value of 0.832, then Factor 3 with ICC coefficients value of 0.771. Factor 6 with ICC coefficients value of 0.758, Factor 2 with ICC coefficients value of 0.750 and Factor 9 with ICC coefficients value of 0.748. The lowest of the 11 factors was Factor 11 with a value of 0.354. The internal consistency and Cronbach’s α values for each factor, as well as the ICC coefficient values for the entire 11 factors of the ATTS are detailed in (Table 2).

## Discussion

The objective of this study was to report the reliability and validity of a Korean version of the ATTS, administered to a sample of university students. EFA was performed on the Korean version to yield 11 factors including 32 items, but CFA failed to support 11-factor model of the scale. Six out of 11 factors were acceptable in terms of both internal consistency and test–retest reliability.

There are disagreements in many factors except few factors on EFA results of the Korean version and the original version of the ATTS [16]. For example, factor 11 (Relation-caused) of the Korean version has a total of two similar items to those of the original version. An intriguing one of such differences is the case that in the original version ‘suicide a right’ and ‘suicide regarding the case of an incurable disease’ included a same factor while they were separated in the Korean version. The reason for that is because western countries have had euthanasia debates in popular for a long time while South Korea have rarely made public discussions over this topic [25].

On the CFA after EFA, the Korean version was not fitted to 11-factor model. This result is similar to an Iranian and a Japanese study which performed CFA whether ATTS translated by their own language to fit 10-factor model [10, 15]. Various models based on the number of factors (2–15) of SOQ also have not satisfied to meet the demand levels of construct validity indices on CFA [3]. One of major reasons why those scales for ATTS have not a stable latent structure would be because items regarding fact and attitudes mixed in a same measure [3].

Although the Korean version has not been proved to have good construct validity in this study, 6 factors (Factor 1–6) on EFA results have acceptable content validity and reliability. For example, all items of factor 2 have concerned with ATTS in incurable diseases case. It means that each factor approximately have acceptable content validity. In particular, six factors (factor 1 ~ 6) have acceptable internal consistency and test–retest reliability, using the criteria of 0.5 as an acceptable minimal level of Cronbach’s α and using that ICC were interpreted with the following criteria: ICC <0.4, poor; 0.4< ICC <0.75, fair or good, ICC >0.75, excellent [26, 27]. If necessary, those factors could be applied in a South Korean setting despite of the risk of poor construct validity on the whole.

However, many researchers emphasize that instrument for the psychometric properties must be multi-dimensional to measure different attitudes areas of human psychology various [27–29]. Furthermore, public attitudes towards suicide in a society would be affected by its own culture and value. In this vein, a tool for attitudes toward suicide, including various dimensions and considering South Koreans’ culture and value, need to be developed. There are some limitations to the interpretation of the results in that the study. For example, target participants was only university students of one university in Seoul. However, this study is the first validity and reliability study of ATTS in South Korea.

## Conclusions

The present study showed that the reliability and validity of ATTS was an acceptable tool to measure ATTS in Korea, despite few factors having very low Cronbach’s α. Thus additional research for reliability is needed.
